# Development of PLA/EVA Reactive Blends for Heat-Shrinkable Film

**DOI:** 10.3390/polym11121925

**Published:** 2019-11-22

**Authors:** Rattikarn Khankrua, Tanyawan Pongpanit, Ponchai Paneetjit, Rungnapha Boonmark, Manus Seadan, Supakij Suttiruengwong

**Affiliations:** 1 Department of Materials Engineering Faculty of Engineering, Rajamangala University of Technology, Rattanakosin 96 Mu 3 Phutthamonthon Sai 5 Road, Salaya, Phutthamonthon, Nakhon Pathom 73170, Thailand; 2 Department of Materials Science and Engineering, Faculty of Engineering and Industrial Technology, Silpakorn University, Sanamchandra Palace Campus, Nakhon Pathom 73000, Thailand; tanyawan2538@gmail.com (T.P.); paneetjit.p@gmail.com (P.P.); boonmark.r@gmail.com (R.B.); 3 Department of Physics, Faculty of Science, Silpakorn University, Sanamchandra Palace Campus, Nakhon Pathom 73000, Thailand; manus_sc.su@hotmail.com

**Keywords:** poly(lactic acid), ethylene vinyl acetate, reactive blend, heat-shrinkable film, mechanical model

## Abstract

Heat-shrinkable films have widely been used for various applications such as shrinkable labels and cap seals. These plastics have generally a short life. The biodegradable polymers can thus be an ideal candidate for such applications. This work aimed to study the stretching and shrinking ratio of poly(lactic acid)/ethylene vinyl acetate through reactive blends system for heat-shrinkable films application. The reactive agents, Joncryl^®^ and Perkadox were used as in situ compatibilizers. PLA/EVA with 100/0, 97/3, 95/5, 93/7, and 90/10 ratios were prepared in the twin screw extruder. Neat PLA and PLA/EVA films were fabricated by blown film extrusion. The results revealed that the elongation at break of PLA in the TD direction was improved when adding EVA. PLA and EVA film with 0.1 phr of Perkadox was found to be sufficient as evident by FESEM micrograph and DMTA results. The films were stretched and shrunk at a temperature of 70 °C. The percentage of shrinkage of the stretched PLA/EVA reactive blend films, two and three times were, 100%, approximately. On the other hand, the four-times stretched films shrunk less than 100% because of the excessive stretching, which resulted in film breakage and defect.

## 1. Introduction

Thermoplastic resins, such as polyethylene, polypropylene, polyethylene terephthalate, and polyvinyl chloride, have been widely used in packaging applications, for example, bottles, containers, vessel, labels, foods wraps, and cap seals, which need shrinkable polymer films. The characteristic of shrink film is its ability upon exposure to heat to either shrink or, if restrained, to create shrink tension within the film. This behavior occurs because the film’s molecules are oriented by rapidly quenching after being stretched and heated to their orientation temperature range. Upon reheating, these oriented molecular segments in the amorphous region start to relax and return to their original random coil state. Therefore, when the wrapped product is passed through a hot air, it causes the film to shrink around the product producing a tight pack [1]. The manufacture of shrink films usually use multilayer films because they allow a combination of their properties, such as shrink tension, optical clarity, hot seal strength, and shrink temperature range, which are unobtainable in a monolayer film. In addition, film multilayering processes result in high-speed packaging lines [2]. However, these processes are relatively expensive due to the cost of the interpolymer, which is used as a compatabilizing layer [3,4]. Heat shrink films conventionally used for packaging and the like are mainly polyvinyl chloride [5,6], polyethylene terephthalate [7,8] polystyrene [9], or polypropylene [10]. These kinds of plastic products are usually used and disposed immediately after use. Lately, many countries have attempted to identify and reduce, or even ban some single-use plastic items as this take-make-use-dispose is not sustainable. The replacement of fossil-based plastics with renewable-based plastics is one of the sustainability options. The biodegradable polymers have currently been utilized as alternative materials to the existing commodity plastics in some certain applications. Among the biodegradable and compostable plastics, poly(lactic acid) (PLA) is one of the most market-available biodegradable polymer. It possesses good mechanical properties as well as superior optical clarity. However, to be suitable for some applications, it requires an improvement in its elongation at break, impact strength, and film flexibility while maintaining its transparency. In addition, PLA film fabricated by blown film extrusion is very difficult to produce due to its brittleness and low melt strength. One of the efficient methods for the PLA film development is the blending with flexible or soft polymers. Ethylene vinyl acetate (EVA) is a copolymer of ethylene and vinyl acetate (VA). The properties of EVA copolymer usually depend on concentration of VA. EVA copolymer with low VA content (<20%) are usually employed as thermoplastics, whereas those with high levels of VA are generally considered as oil-resistant elastomers [11,12]. Several works have been carried out for PLA/EVA blends and reported that the incorporation of EVA into PLA significantly improved the elongation at break, toughness, and impact strength of PLA [13,14,15,16,17]. Moreover, the addition of reactive agents could further improve the compatibility between polymers, for instance, glycidyl methacrylate [18], phthalic anhydride [19], and peroxide [20,21]. The addition of reactive agents could promote in situ copolymerization through the free radical reaction using a peroxide initiator. The interfacial adhesion between phases of two polymers can be consequently improved.

To the best of our knowledge, the systematic reactive blend of PLA/EVA including the mechanical model of the stretching and shrinking behavior of PLA/EVA reactive blend films have not been reported. Therefore, this work aimed to investigate the mechanical properties and thermal properties of PLA/EVA in reactive blend systems. The addition of EVA could lead to an increase in the blown film extrusion capability of PLA. The heat shrinkable behavior of PLA/EVA reactive blends film was also studied and discussed.

## 2. Materials and Methods

### 2.1. Materials

The grade LX-175 PLA was kindly supplied by Total Corbion PLA (Bangkok, Thailand) Ltd. The melting and the glass transition temperatures were 155 and 55 °C, respectively. The grade TV-1055 EVA, having a vinyl acetate content of 28%, was purchased from TPI Polene (Public) Co., Ltd. (Bangkok, Thailand). The Joncryl^®^ 4368 was obtained from BASF, Bangkok, Thailand. Its molecular weight (*M*_w_) was 6800 Da with the epoxy equivalent weight of 285 g/mol. Di(tert-butylperoxyisopropyl) benzene or Perkadox14s (peroxide 38.64%) was purchased from AkzoNobel, Bangkok, Thailand.

### 2.2. Processing of PLA/EVA Film

PLA and EVA pellets were first dried at 60 °C for 6 h before use. The formulations and compositions of PLA/EVA blends were showed in the Table 1. The amount of Perkadox (designated as P) was varied from 0.1 to 0.2 phr whereas Joncryl^®^ (referred to J) was mixed as a viscosity modifier with the fixed concentration at 0.5 phr. PLA/EVA and reactive agents were melt-mixed in a twin screw extruder under a temperature profile from the feed zone to the die set of 80/110/150/170/180/180/190/190/190/190 °C with a screw speed of 450 rpm. The extruded PLA/EVA blends were dried, cut, and kept in a plastic zip bag. Neat PLA and PLA/EVA with reactive agent films were fabricated through extrusion blowing under a temperature profile from the feed zone to the die set of 135/165/175/185/190 °C with the screw speed of 45.5 rpm. A film-blowing tower was collected with a calendering nip and take-up rolls.

### 2.3. Characterization and Testing of Films

The melt flow index of all samples was performed on a melt flow indexer operating at 190 °C with a 2.16 kg load according to ASTM D1238.

The mechanical properties of the blend films were tested by using a universal testing machine (Instron 5969, Instron, MA, USA) in accordance with ASTM D882. The samples were cut into strips from blown films and divided in two directions; the machine direction (MD) and transverse direction (TD). The strips were tested at a cross-head speed of 50 mm/min.

The dynamic mechanical thermal properties of PLA/EVA blend were examined using ANTON PAAR, modular compact rheometer (MCR302, Anton Paar, Graz, Austria) equipped with rectangular fixture (SRF) holders. A temperature sweep was heated from 30 to 150 °C at a heating rate of 3 °C/min under the dynamic mechanical thermal analysis (DMTA) torsion mode at a frequency of 1 Hz. To prepare the DMTA specimen, the extruded PLA/EVA was compressed by compression molding and the sample were laser-cut to dimensions of 10 mm × 1.16 mm × 40 mm.

To investigate the reaction between PLA and EVA, the gel content of PLA/EVA film was measured. Sample films were dissolved in chloroform at 60 °C for 24 h. The gel content was computed by the following Equation (1):

(1) $$\mathrm{Gel}\;\mathrm{content}\;(\%)\;=\;\left(\frac{w_{g}}{w_{0}}\right)\;\times \;100$$

where w_0_ is the original weight (dry) of the samples, and w_g_ is the weight remaining (dry gel component).

To follow the possible chemical reaction of PLA and EVA, Fourier transform infrared spectroscopy (FTIR) was performed at ambient temperature on a Vertex 70 spectrometer (Bruker, MA, USA). Before testing, the dried gel from the gel content experiments were mixed with potassium bromide (KBr) into flakes. The FTIR spectra of the samples were recorded in the wavenumber range of 400–4000 cm^−1^.

### 2.4. Stretching and Shrinking Tests

To study the stretching and shrinking behavior of films, the films of PLA/EVA with reactive agents were stretched by a uniaxial stretching machine at a laboratory scale. The film samples were prepared with a size of 5.08 cm × 12.4 cm. The grid lines of a 4 × 8 tables with 1 cm × 1 cm cells were drawn on the films surface for further shrinkage measurement. The films were held and stretched by the uniaxial stretching machine with stretching ratios of 2, 3, and 4 under a temperature of 70 °C. Then the stretched films were cooled immediately to preserve the stretched films’ shapes and sizes. After that the stretched films were reheated by introducing to a water bath at 70 °C for 1 min to create the stretched film shrinkage. The dimensions of films after soak in the water bath were measured and the percentage of shrinkage in the machine direction (MD) and the transverse direction (TD) was calculated by Equation (2):

(2) $$\%\;\mathrm{shrinkage}\;=\;\frac{{(L}_{\mathrm{stretched}}{-\;L}_{\mathrm{shrunk}})}{{(L}_{\mathrm{stretched}}{-L}_{i})}\times \;100$$

where L_shrunk_ is the length of the sample after it was shrunk, L_stretched_ is the length of the sample after stretching and L_i_ is the initial length of the sample.

The morphology of blends and PLA/EVA reactive blend films were investigated by field emission scanning electron microscopy (FESEM) (TESCAN MIRA3 LMH Schottky, Brno, Czech Republic). The samples were fractured under liquid nitrogen. The samples were gold-coated before studying the morphology.

## 3. Results and Discussion

### 3.1. Melt Flow Index of Neat PLA and PLA/EVA Blends

The melt flow index values (MFI) of all samples were listed in Table 2. The MFI values of PLA/EVA increased with the EVA contents. The MFI values of Joncyl or Perkadox added to the PLA/EVA blend decreased when compared to that of neat PLA, indicating the higher viscosity and the possible reaction between PLA or EVA and incorporated reactive agents. The addition of Joncryl into the PLA/EVA blend could result in the coupling reaction between end groups of PLA and multifunctional epoxide groups of Joncryl, which consequently led to the higher molecular weight and viscosity of PLA [22,23]. On the other hand, the Perkadox could promote the free radical reaction between PLA and EVA, hence leading to the improved interfacial adhesion between PLA and EVA. The viscosity was thus increased as a result of the higher molecular weight or copolymer. The possible reaction cross-linked PLA and EVA is proposed and discussed later.

### 3.2. Morphology and Fracture Surface of PLA/EVA Blend

**Figure 1:**
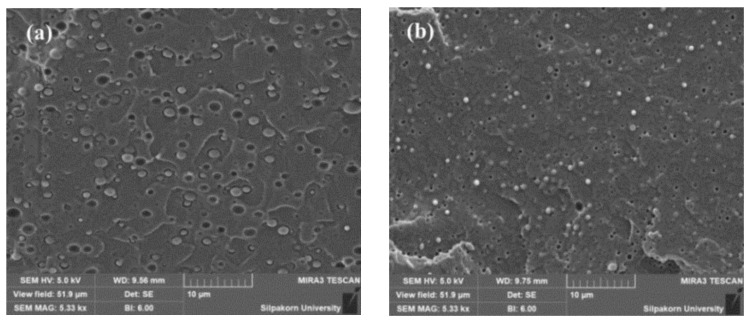
Morphology of (**a**) PLA95 and (**b**) PLA95 + J0.5.

The morphology of blends and PLA/EVA reactive blend films were investigated by field emission scanning electron microscope (FESEM). From SEM micrograph of PLA/EVA reactive blends in Figure 1, it was shown that the dispersion of EVA particles within the PLA matrix with the dimension of EVA dispersed phase of approximately 2 μm. The voids between PLA matrix and dispersed EVA particles could be observed in non-reactive blend (Figure 1a). It indicated the poor interface of PLA and EVA. In the case of adding Joncryl to the blend (in Figure 1b), the good and fine dispersion with the smaller particle size of EVA could be seen. This implied that the interfacial adhesion between PLA and EVA phases was improved by the reaction between epoxide groups and PLA or EVA chains.

**Figure 2:**
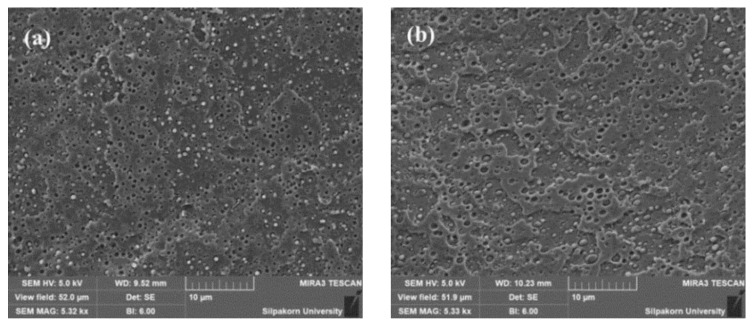
Morphology of (**a**) PLA90 + J0.5/P0.1 and (**b**) PLA90 + J0.5/P0.2.

For the addition of Perkadox, the SEM micrographs of PLA90 + J0.5/P0.1 and PLA90 + J0.5/P0.2 were showed in Figure 2a) and Figure 2b, respectively. The addition of Perkadox at 0.2 phr exhibited the larger EVA particles and non-uniform size when compared to 0.1 phr. This attributed to the possible increasing of the cross-link reaction and led to the poor interfacial adhesion between PLA and EVA phases. This result agreed with the gel content and DMTA results discussed in the next section.

**Figure 3:**
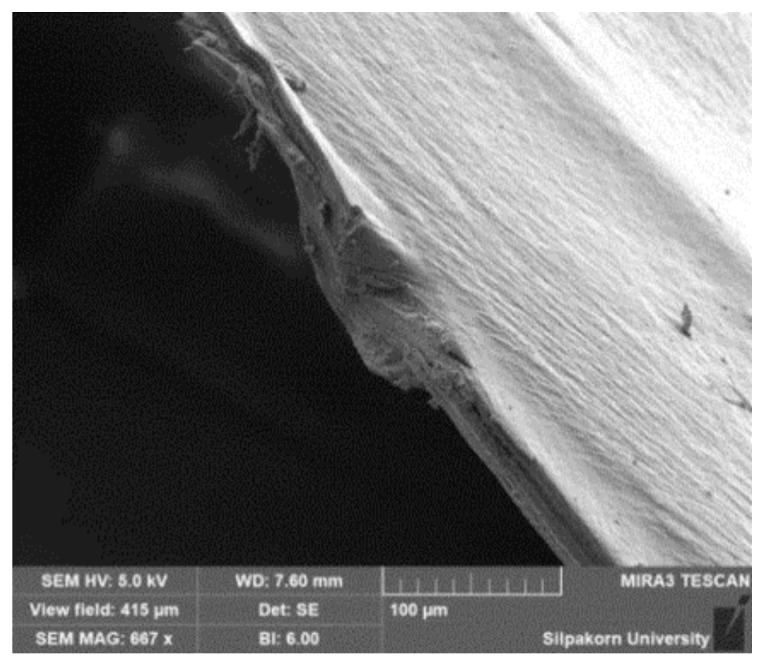
Fracture surface of PLA90+J0.5/P0.2.

Furthermore, for the tensile properties of PLA/EVA reactive blend, it was expected that the increased Perkadox content from 0.1 to 0.2 phr would increase the elongation at break but the elongation at break of PLA90 + J0.5/P0.2 was lower than expectation. Therefore, the fracture surface of tensile testing was investigated. The result showed that SEM micrograph of fracture surface of PLA90 + J0.5/P0.2 (in Figure 3) exhibited the dent on the surface of film. This might be due to the gel on the film and could act as a stress concentrator and initiate the crack and defect, thus lowering the elongation at break.

### 3.3. Gel Content

The gel contents were determined in order to understand the role of the reactive agents on the blend system. The high gel contents imply the change of the undissolved macromolecules, which can be derived from the network structures or larger molecular weights of the polymers. The gel contents of neat PLA and PLA/EVA reactive blend films is summarized in Table 3. The results show that the addition of Joncryl in PLA leads to an increase in the gel content from 0.33% to 2.78%. This is because Joncryl acts as a chain extender which reconnects the PLA chains [23]. Consequently, the larger macromolecules and the higher molecular weight of PLA structures were formed. This effect was less when EVA was added for all content. Moreover, the gel contents increased with Perkadox and EVA contents. The higher peroxide concentrations could promote higher degree of the cross-links.

**Figure 4:**
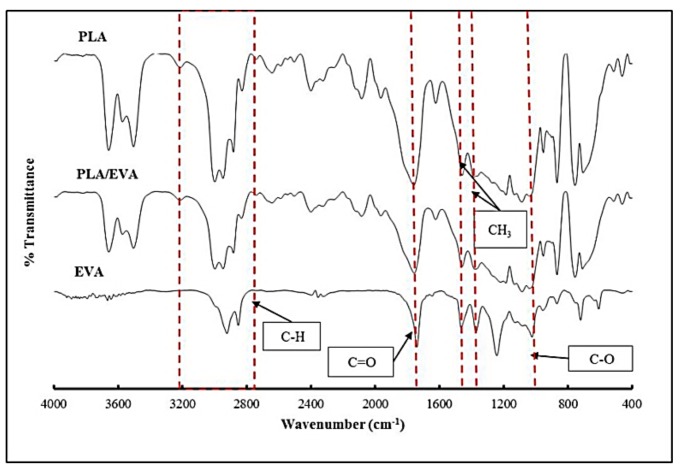
FTIR spectra of gel of neat PLA, PLA/EVA, and neat EVA.

The gel residues from the gel content experiments were subjected to the FTIR spectroscopy characterization. The FTIR spectra of gels obtained from neat PLA, PLA/EVA without reactive agents and neat EVA are showed in Figure 4.

**Figure 5:**
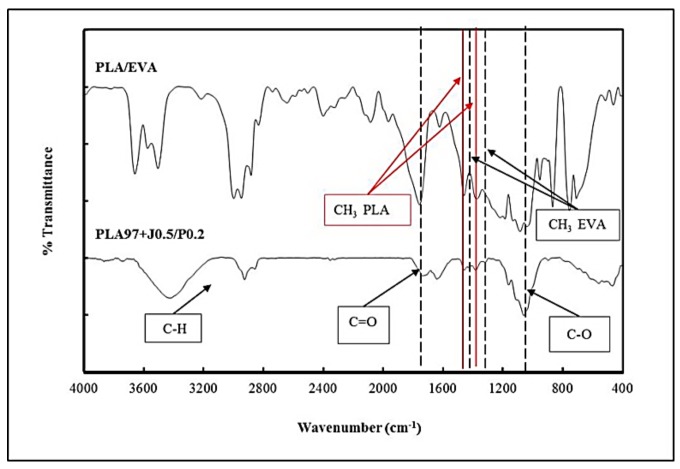
FTIR spectra of gel of PLA/EVA compared to gel of PLA97 + J0.5/P0.2.

The peak signal at 1759 cm^−1^ refers to the carbonyl group (C=O) of the ester group of PLA. The peak appearing at 1087 cm^−1^ belongs to the ester bond (C–O–C). The peaks at 2978 and 2947 cm^−1^ are assigned to C–H stretching. The peaks at 1458 and 1385 cm^−1^ are assigned to the bending of –CH_3_. For EVA, the peaks at 2923 and 2852 cm^−1^ are assigned to C–H stretching. The peak at 1740 cm^−1^ is assigned to C=O stretching. The peak located at 1043 cm^−1^ refers to C–O stretching, and the peaks at 1463 and 1372 cm^−1^ correspond to the bending of –CH_3_. Furthermore, we were unable to detect peak shifting in the PLA/EVA without the reactive agents’ spectrum. This could mean that there were no reactions between PLA and EVA for non-reactive blend. The comparison of the FTIR spectra between PLA/EVA without reactive agents gel and PLA97 + J0.5/P0.2 gel are shown in Figure 5. The result reveals that the FTIR spectrum of PLA97 + J0.5/P0.2 gel exhibits characteristic peaks both of neat PLA and neat EVA. In addition, it was observed that the peak at 1736 cm^−1^, which refers to the carbonyl group (C=O), is shifted to a lower wavenumber. This suggests evidence of the reaction.

**Figure 6:**
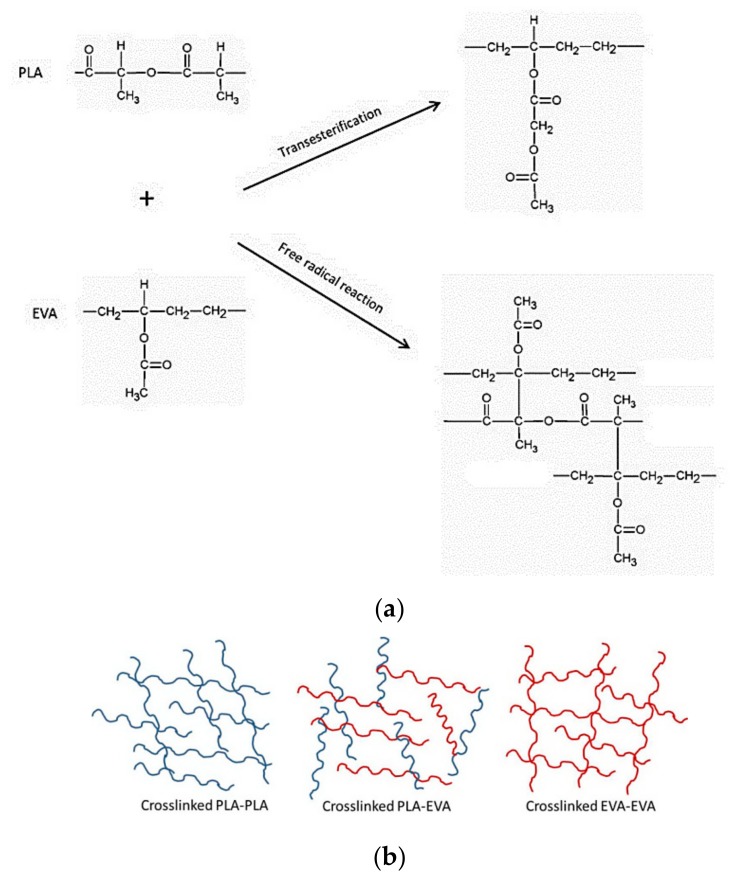
(**a**) Possible reaction between PLA and EVA and (**b**) the reaction scheme for PLA/EVA reactive blend.

The reaction between PLA and EVA has been reported elsewhere [24,25] and could occur through a transesterification reaction in the case of addition with catalyst [25]. In this work, the reaction between PLA and EVA could arise from the free radical reaction. The free radical produced by the decomposition of Perkadox abstracted the hydrogen in both PLA and EVA, leading to free radical generation in PLA and EVA. This free radical via hydrogen abstraction led to the cross-linked structures between PLA and EVA. The possible reactions between PLA and EVA is presented in Figure 6a. In addition, the free radical reaction products could be not only PLA–EVA cross-linking but also EVA–EVA and PLA–PLA cross-linkings [24,26] as shown in Figure 6b.

### 3.4. Tensile Properties

**Figure 7:**
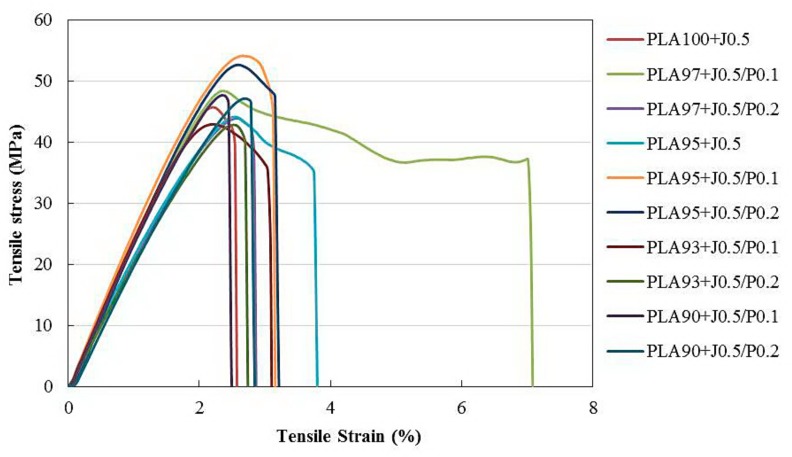
Stress-strain curves of PLA/EVA blend film in machine direction (MD).

**Figure 8:**
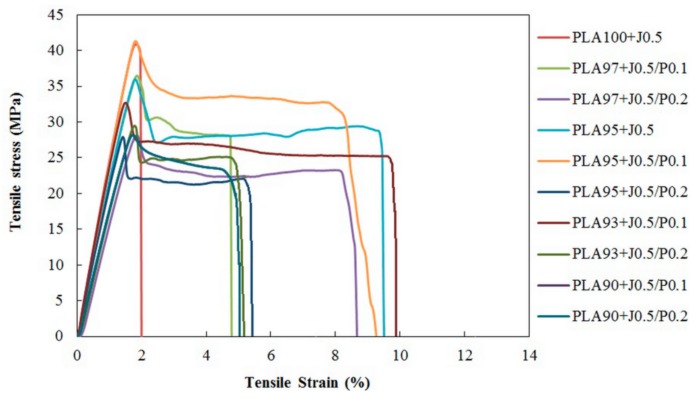
Stress-strain curves of PLA/EVA blend film in transverse direction (TD).

The film samples were prepared using a blow film extrusion. In this case, the fixed amount of multifunctional epoxides was added to all samples in order to improve the blowability and the melt viscosity. The tensile properties of blend films were tested in both machine direction (MD) and transverse direction (TD). The stress-strain plots of both directions were showed in Figure 7 and Figure 8, respectively. Since neat PLA film was not smooth and there were a large number of creases on the film due to its low melt strength, it was not suitable for blown film extrusion. Therefore, neat PLA film was not introduced to the test. It was obvious that EVA addition caused the film fabrication enhancement, hence, PLA/EVA blend films can be produced and tested. It can be seen in Figure 7 that the elongation at break of PLA/EVA blends are comparable to PLA100 + J0.5, except the PLA97 + J0.5/P0.1 sample exhibited the highest toughness film in the machine direction (MD). Even though the elongation at break of PLA/EVA reactive blend films was insignificantly improved in MD, but more importantly, PLA/EVA reactive blend films were successfully blown. In addition, the *tensile* results of the blend films with the transverse direction (TD) obviously illustrated the plastic deformation for PLA/EVA blend. This indicated that the addition of EVA could improve the elongation at break of PLA. PLA93 + J0.5/P0.1 showed the highest elongation at break in the transverse direction.

**Figure 9:**
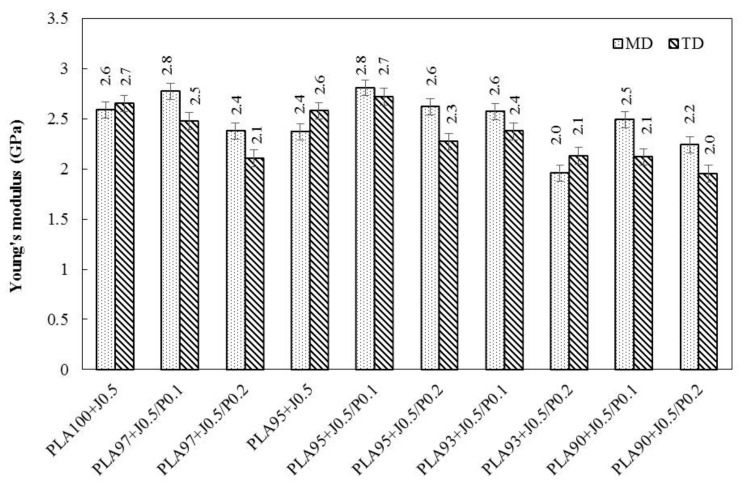
Young’s modulus of PLA/EVA blends film in MD and TD direction.

**Figure 10:**
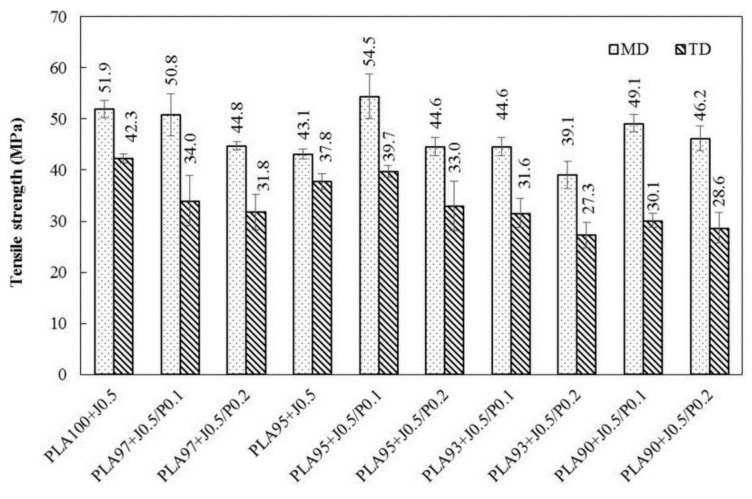
Tensile strength of PLA/EVA blends film in MD and TD direction.

**Figure 11:**
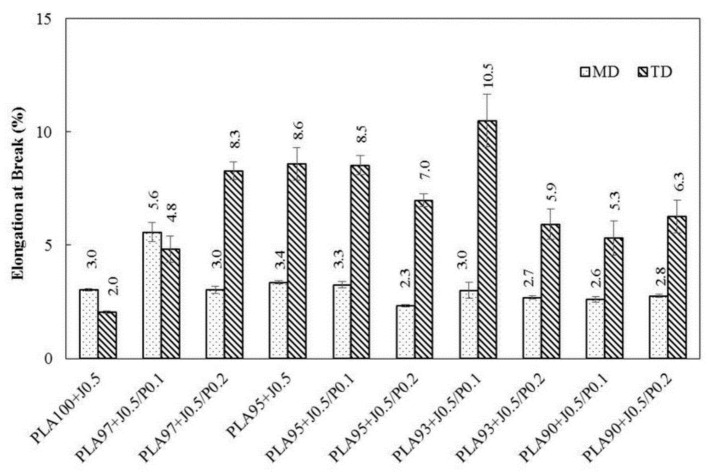
Elongation at break of PLA/EVA blends film in MD and TD direction.

Young’s modulus, tensile strength, and elongation at break of all blend films are summarized in Figure 9, Figure 10 and Figure 11, respectively. Considering the Young’s modulus of the blend films (in Figure 9), it could be seen that the Young’s modulus of blend films in the MD direction were slightly higher than that of the TD direction. The addition of 5 wt % EVA did not significantly affect the Young’s modulus of the blend films. With a further increase of EVA concentration, the Young’s modulus slightly decreased because EVA acts as a soft and tough phase. Young’s modulus of PLA95 + J0.5/P0.1 which composed of 5 wt % EVA was 2.8 GPa. It decreased to 2.6 GPa for PLA93 + J0.5/P0.1. Moreover, the addition of Perkadox with 0.1 phr could increase the Young’s modulus. This could be attributed to the cross-link reaction between PLA and EVA promoted by peroxide. However, when the content of Perkadox increased from 0.1 to 0.2 phr, the Young’s modulus of samples tended to decrease.

Considering the tensile strength of blend films in MD compared to TD as shown in Figure 10, the tensile strength of the MD films were higher than those of the TD films because the molecules of polymers oriented along the blowing direction or machine direction. When the Perkadox concentration increased from 0.1 to 0.2 phr, the tensile strength of the blend films tended to decrease. PLA90 + J0.5/P0.1 film exhibited a tensile strength in MD of 49.1 MPa. When Perkadox was increased to 0.2 phr, PLA90 + J0.5/P0.2 film, the tensile strength decreased to 46.2 MPa. This might be caused by the higher cross-link degree and the stress concentration in the blends [27].

The elongation at break of the TD films shown in Figure 11 was higher than those of the MD films. This could result from the low frost line in the blown film process and cause the partial orientation of the polymer’s molecules along the machine direction in blend films while the others could orient in the radial or transverse direction. Furthermore, the elongation at break of the TD films increased with EVA contents. However, when the content of EVA was increased to 5%, the increase of Perkadox content led to the lower elongation at break, in which the decrease was seen from 8.5% to 7.0% for PLA95 + J0.5/P0.1 and PLA95 + J0.5/P0.2 films in TD, respectively. This may be attributed to the higher degree of cross-links evolved during the free radical reaction and may be a result of the reaction between EVA itself, leading to the larger the formation of cross-linked EVA particles. These might, consequently, be attributed to the poor interfacial adhesion between PLA and EVA phases.

### 3.5. Effect of EVA and Perkadox Contents on Dynamic Mechanical Thermal Properties of PLA/EVA Blend

**Figure 12:**
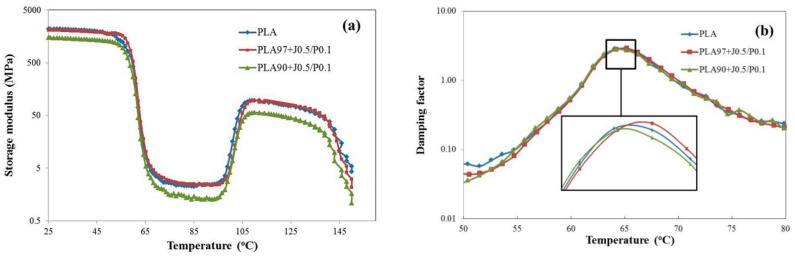
The effect of EVA content on dynamic mechanical thermal properties of PLA/EVA blend. (**a**) Storage modulus and (**b**) damping factor with an enlarged inset at *T*_g_.

The effect of EVA and Perkadox contents on the dynamic mechanical thermal properties of PLA/EVA blends was investigated. The dependence of the storage modulus on the temperature of neat PLA and PLA/EVA reactive blends were showed in Figure 12a. To clarify the effect of EVA contents on mechanical thermal properties of PLA/EVA blends, the PLA/EVA reactive blends with 3 and 10 wt % of EVA were chosen for comparison. From Figure 12a, it could be observed that neat PLA showed the high storage modulus in the glassy region (25–50 °C) with the storage modulus of around 2200 MPa. The storage modulus of PLA/EVA reactive blends decreased when the EVA contents increased. Further, at temperature above 60 °C, the storage moduli of PLA and blends were significantly declined. At the temperature above *T*_g_, PLA and EVA are in a rubbery state. In the rubbery region, blending with EVA 10 wt % showed the lower the storage modulus when compared to neat PLA and blending with EVA 3 wt %. This was attributed to the addition of soft and tough EVA phase in the blends, which enhanced the impact load transfer and led to the toughening of the blends [14]. Then, the storage moduli of neat PLA and blends were increased again after 100 °C. This phenomenon was due to the nature of PLA. The increase in the storage modulus is a consequence of the cold crystallization of PLA in the test specimens during the DMTA temperature scan, which was also reported in other studies [28,29,30].

In addition, upon increasing the EVA content to 10 wt %, the glass transition temperature (*T*_g_) of the PLA/EVA reactive blend was slightly shifted to the lower temperature (as shown in Figure 12b). This was caused by the plasticizing effect of EVA in PLA matrix when the temperature increased.

**Figure 13:**
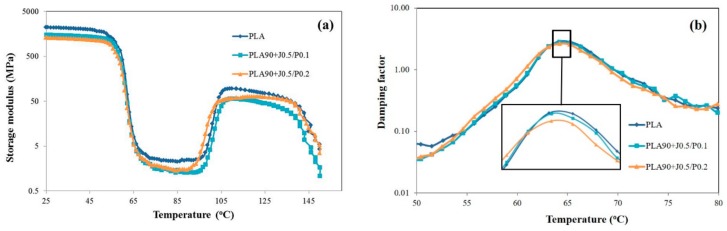
The effect of Perkadox content on dynamic mechanical thermal properties of PLA/EVA blend. (**a**) Storage modulus and (**b**) damping factor with an enlarged inset at *T*_g_.

The effect of the Perkadox contents on the dynamic mechanical thermal properties of PLA/EVA blend is shown in Figure 13a. The storage modulus of PLA/EVA reactive blends decreased with the increase in Perkadox content. This result is in agreement with Young’s modulus values obtained from the tensile testing. Figure 13b shows a plot of damping factor against the temperature for PLA, PLA90 + J0.5/P0.1, and PLA90 + J0.5/P0.2. The damping peak height of PLA90 + J0.5/P0.1 was slightly higher than that of PLA90 + J0.5/P0.2. This could imply that Perkadox might induce the reaction between PLA and EVA phases and resulted in strong bond interaction in PLA/EVA blends [24]. During the dynamic mechanical thermal testing, the interaction bonds dissociated and reformed, consuming a great deal of energy. Consequently, the loss factor increased and the damping properties improved [31]. This indicated that the interaction between phases of PLA/EVA reactive blends with Perkadox 0.1 phr was better than that of Perkadox 0.2 phr. This result is in agreement with the FESEM images.

### 3.6. Model of Stretching and Shrinking Behavior of PLA/EVA Reactive Blend

The polymer which possesses the capability to recover to its original shape after application of an external stimulus (e.g., light, heat, pH, etc.) is called a shape memory polymer (SMP). The primary mechanism of shape memory in polymer is related to the low entropy state during deformation from an original shape above the transition temperature (*T*_trans_). Shape recovery occurs and returns to a high entropy state when the polymer is reheated above the *T*_trans_. The *T*_trans_ can be either a glass transition temperature (*T*_g_) or a melting temperature (*T*_m_) of the polymer [32,33]. In addition, the shrinkage of amorphous polymers can be also described by a viscoelastic model.

**Figure 14:**
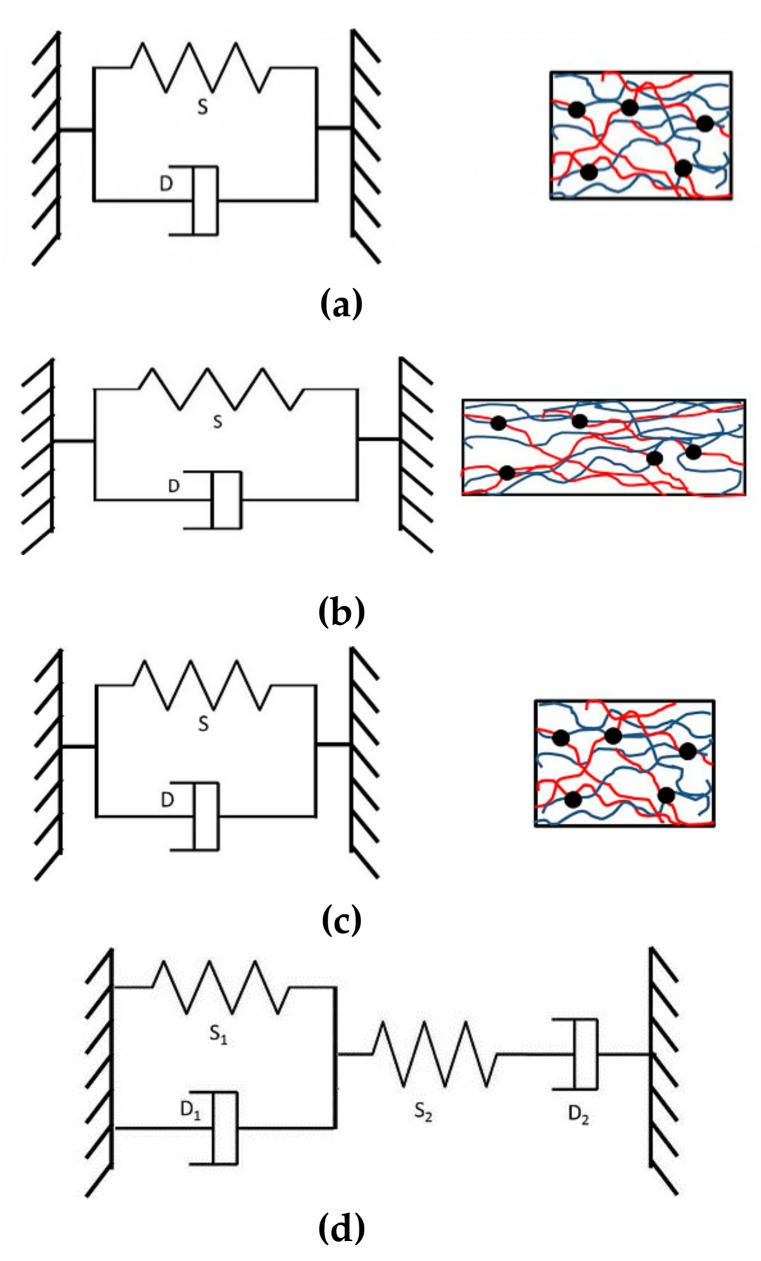
The proposed mechanical model with molecular mechanism for stretching and shrinking behavior of PLA/EVA reactive blend. The red line, blue line and black dot were PLA chains, EVA chains and crosslink point between PLA and EVA chains, respectively. (**a**) The proposed mechanical model; (**b**) heating-stretching-cooling; (**c**) reheating and shrinking; and (**d**) the partial shrinkage mechanical model.

The viscoelastic materials can exhibit both viscous and elastic behavior. The deformation of liquid materials is illustrated by a dashpot. The elasticity of the solid materials is represented by a spring. Therefore, the viscoelastic materials can be described by the combining spring and dashpot. The mechanical model and molecular mechanism to explain stretching and shrinking behavior of PLA/EVA with reactive agents were shown in Figure 14a. The model consisted of a spring (S) and a dashpot (D) connected in parallel. It is noted that the solid circle in molecular mechanism figures indicated the cross-linked points within the blend structure. When the film was stretched at the temperature above its *T*_g_, the spring and a dashpot were stretched easily because of the low viscosity of the dashpot (Figure 14b). At this temperature, the chain segments were flexible. After that, the stretched film was cooled immediately and created the increase of the dashpot viscosity resulting in the internal stress increase in the extended spring. This process causes the polymer film to memorize its original shape. The flexibility of the entire segment was limited. Furthermore, the temporary entanglement of polymer chains and the cross-link points can be used for the fixation the stretched shape. Then, the stretched film was reheated, causing the decrease of dashpot viscosity. Hence, the spring started to contract to its original track by the force from retained stress (Figure 14c). Thus, the temporary fixed polymer chains recoiled back to its original dimension. However, this model represented only the case of fully shrinking film. The mechanical model of the film where the shrinkage did not reach 100% could be described by using the modeling of polyester [34]. This simple model combines a unit of a spring S_1_ and a dashpot D_1_ connected in parallel, and a unit of the spring S_2_ and the dashpot D_2_ connected in series, as shown in Figure 14d. After re-heating, the dashpot D_2_ started to relax and extend because of the contraction from the S_1_–D_1_ parallel unit. Therefore, the series unit cannot shrink to its original position, thus causing partial film shrinkage.

**Figure 15:**
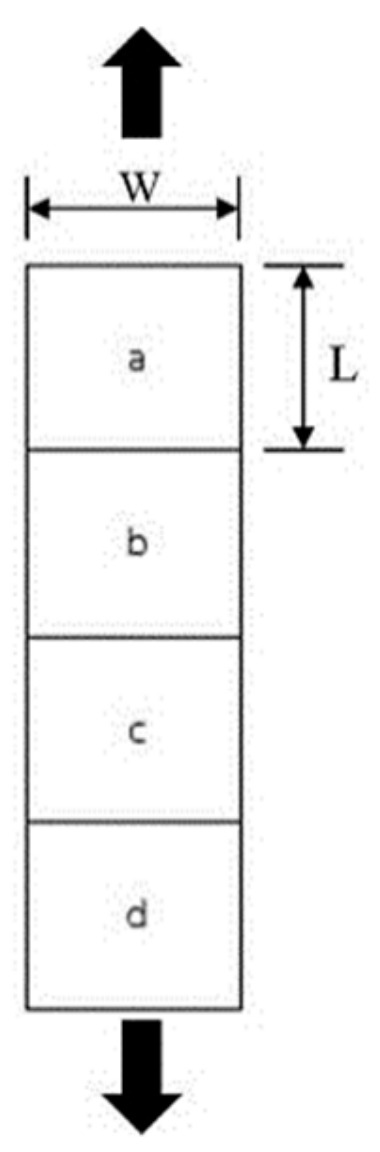
Film specimen for heat shrinkage testing and stretching direction.

In this part, the stretching of PLA/EVA with reactive agent films was prepared by a uniaxial stretching machine at 70 °C. The film specimens were prepared as shown in Figure 15. The a, b, c and d were the four different zones of film specimen. The percentage of film shrinkage was measured from two dimensions (the length (L) and the width (W)) based on the length difference before and after reheat. The average percentage of the shrinkage of PLA/EVA blend films at different stretching ratios of 2 and 3 for both MD and TD directions are shown in Table 4 and Table 5. The results revealed that the percentage of film shrinkage in the MD films was comparable to the TD films. For the films stretched with stretching ratios of 2 and 3, the shrinkage percentage reached 100%. However, with a stretching ratio of 4, the shrinkage of the film tended to decrease.

Considering the effect of EVA contents on the shrinkage behavior, it was found that when EVA contents was increased in blend from 3% to 5% by weight (at a fixed content of reactive agents), those percentages of shrinkage were comparable. Therefore, to clarify the effect of EVA content on the percentage of shrinkage, EVA amounts of 3 and 10 wt % were chosen for the sake of the comparison. When EVA contents increased to 10 wt %, the percentage of shrinkage was higher when compared to the PLA blend with EVA 3 wt %. This result was similar for the stretching ratios of 2 and 3 (as shown in Table 4 and Table 5), except at a stretching ratio of 4. The stretching ratio of 4 led to a strain greater than the yield point of the polymer blend films, hence, it could not return to its original length.

From the mechanical model as explained previously, springs were used to describe the reversible deformation of the materials. Hence, the spring modulus could represent the material’s modulus. From the tensile results, it could be seen that the addition of EVA in PLA led to a lowering in the Young’s modulus of the blends. This could mean that EVA reduced the spring modulus of the blends; therefore, the PLA/EVA film blend easily deformed and returned to its original length. Moreover, the amount of shrinkage depends on the concentration and orientation of the oriented and disoriented amorphous phases which are the predominant mechanisms [32,35]. The increase of EVA content gives the increase of the amorphous phase. Therefore, the percentage of the shrinkage of films increased with EVA content. Furthermore, the increases of Perkadox content caused the percentage of shrinkage to increase for both of MD films and TD films with EVA content of 3, 5, and 7 wt %. The degree of shrinkage was controlled by the concentration and orientation of the amorphous phase [32] as well as the memory points depending upon cross-links [36,37]. Cross-link points help in the shrinking process by serving as a memory in the stretched sample. They revert to their original positions during shrinkage [38]. Consequently, a higher cross-linking reaction between the polymer molecules was of great advantage to the shrinking mechanism.

However, the blend film with 10 wt % of EVA exhibited the decreases of percentage of films shrinkage when the Perkadox content increased. The highest of percentage of film shrinkage was observed in PLA90 + J0.5/P0.1. In the case of the stretching ratio of 4, the increase in the percentage of shrinkage with Perkadox content were observed in blends at EVA amount of 3 and 5 wt %. Then it decreased with an increase of EVA higher than 5 wt %. The low percentage of shrinkage could be observed from the formula that exhibited the high gel content (2.72% for PLA90 + J0.5/P0.2). The increase of EVA content led to the increase of gel content. Due to the greater number of tertiary type of carbons, the chance of cross-linking reaction increases [39]. This might be due to the presence of gel in the film which could obstruct the shrinkage of the film.

When comparing the percentage of shrinkage in the length and the width directions of the same specimen, it could be seen that the shrinkage in the L direction was higher than the W direction. This is because, when the films were stretched along the L direction, the polymer chains of the films oriented along the stretched direction and had a better return to the original random coil state in the L direction than the W direction while reheating. In addition, the shrinkage of the film was obviously observed at a and d positions because they were the edge of the specimen film. Consequently, they were easier to shrink than the middle position.

### 3.7. Morphology of Stretched and Shrunk of PLA/EVA Reactive Blend Films

**Figure 16:**
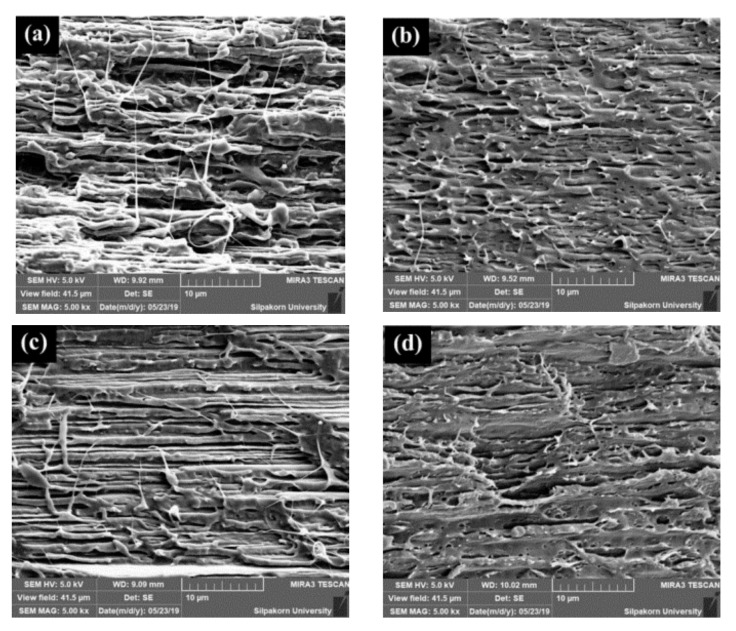
The morphology of the cross-section in TD of (**a**) stretched PLA90 + J0.5/P0.1 film, (**b**) stretched PLA90 + J0.5/P0.2 film, (**c**) shrunk PLA90 + J0.5/P0.1 film, and (**d**) shrunk PLA90 + J0.5/P0.1 film.

**Figure 17:**
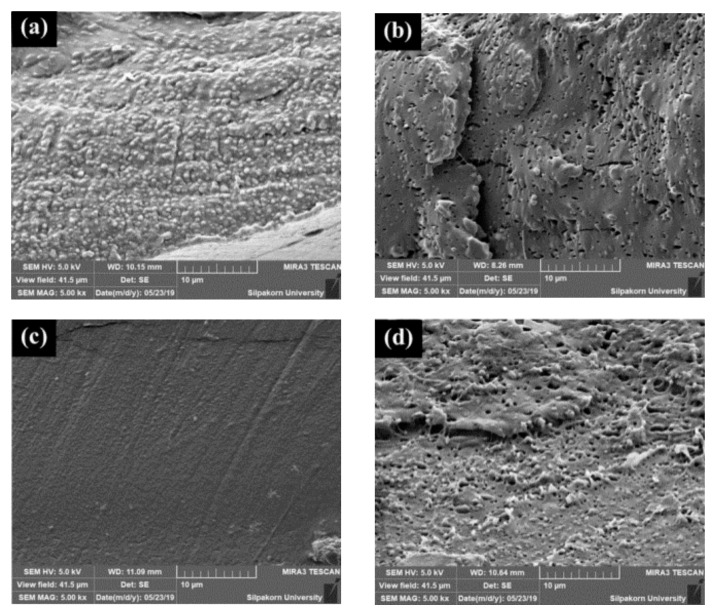
The morphology of the cross-section in MD of (**a**) stretched PLA90 + J0.5/P0.1 film, (**b**) stretched PLA90 + J0.5/P0.2 film, (**c**) shrunk PLA90 + J0.5/P0.1 film, and (**d**) shrunk PLA90 + J0.5/P0.1 film.

The morphology of the cross-section of stretched and shrunk PLA/EVA reactive blend films in TD and MD are illustrated in Figure 16 and Figure 17, respectively. It can be seen in Figure 16 that TD films displayed a stack morphology. This characteristic is from the perpendicularly film preparation direction when film was blown. In addition, the PLA90 + J0.5/P0.1 stretched film (Figure 16a) showed larger gap between layers than the PLA90 + J0.5/P0.2 stretched film (Figure 16b). However, the PLA90 + J0.5/P0.1 film exhibited a better stacking pack than the PLA90 + J0.5/P0.2 film after films shrinking. The shrink film morphology of PLA90 + J0.5/P0.2 sample also showed some restraint between layers that might cause the smaller percentage of shrinkage than the PLA90 + J0.5/P0.1 sample.

The morphology of MD sections (Figure 17) revealed the occurrence of pinholes in the PLA90 + J0.5/P0.2 film. The holes were larger and still located in the film after shrinking. Moreover, the surface roughness was roughly greater in the PLA90 + J0.5/P0.2 sample than in the PLA90 + J0.5/P0.1 sample.

## 4. Conclusions

The brittleness of PLA was improved when EVA was introduced. The addition of EVA led to an increase of blown film extrusion capability of PLA. In addition, the PLA/EVA film with 0.1 phr of Perkadox was observed as the optimal compatibilizer concentration which led to the maximum toughness at PLA/EVA 93/7 and highest modulus at 95/5. The high loading of peroxide could cause the high cross-link degree and phase separation. The percentage of shrinkage of PLA/EVA reactive blends films were 100%, when the films were stretched two and three times of their original length. However, the shrink recovery was less than 100% when the films were stretched up to four times of its original length. The heat-shrinkable behavior of the PLA/EVA reactive blend could be described by the shape memory effect. It can be illustrated as the spring and dashpot connected in parallel regarding to the fully shrinkage film. However, the film where the shrinkage had not reached 100% could be demonstrated by the addition of series connections between the spring and dashpot to the parallel spring-dashpot unit.

## Figures and Tables

**Table 1:** Sample formulations and blend compositions.

	Formula	Compositions			
	PLA + Joncryl/Perkadox	PLA (%)	EVA (%)	Joncryl (phr)	Perkadox (phr)
1	Neat PLA	100	-	-	-
2	PLA100 + J0.5	100	-	0.5	-
3	PLA97 + J0.5/P0.1	97	3	0.5	0.1
4	PLA97 + J0.5/P0.2	97	3	0.5	0.2
5	PLA95	95	5	-	-
6	PLA95 + J0.5	95	5	0.5	-
7	PLA95 + J0.5/P0.1	95	5	0.5	0.1
8	PLA95 + J0.5/P0.2	95	5	0.5	0.2
9	PLA93 + J0.5/P0.1	93	7	0.5	0.1
10	PLA93 + J0.5/P0.2	93	7	0.5	0.2
11	PLA90 + J0.5/P0.1	90	10	0.5	0.1
12	PLA90 + J0.5/P0.2	90	10	0.5	0.2

**Table 2:** Melt flow index (MFI) of samples.

Sample	MFI (g/10min)
Neat PLA	18.7 ± 1.5
PLA100 + J0.5	5.0 ± 0.1
PLA97 + J0.5/P0.1	3.9 ± 0.3
PLA97 + J0.5/P0.2	3.9 ± 0.2
PLA95	7.7 ± 0.5
PLA95 + J0.5	3.3 ± 0.3
PLA95 + J0.5/P0.1	6.9 ± 0.3
PLA95 + J0.5/P0.2	7.2 ± 0.3
PLA93 + J0.5/P0.1	5.5 ± 0.4
PLA93 + J0.5/P0.2	5.1 ± 0.1
PLA90 + J0.5/P0.1	2.6 ± 0.3
PLA90 + J0.5/P0.2	3.6 ± 0.3

**Table 3:** Gel content of PLA/EVA reactive blend film.

Formula	Gel Content (%)
Neat PLA	0.33 ± 0.02
PLA100 + J0.5	2.78 ± 0.54
PLA97 + J0.5/P0.1	0.10 ± 0.14
PLA97 + J0.5/P0.2	0.47 ± 0.01
PLA95 + J0.5	2.30 ± 0.03
PLA95 + J0.5/P0.1	0.49 ± 0.04
PLA95 + J0.5/P0.2	1.23 ± 0.09
PLA93 + J0.5/P0.1	0.55 ± 0.13
PLA93 + J0.5/P0.2	1.78 ± 0.52
PLA90 + J0.5/P0.1	0.62 ± 0.44
PLA90 + J0.5/P0.2	2.72 ± 0.20

**Table 4:** The average percentage of shrinkage of film at a stretching ratio of 2 in MD and TD.

Direction	Position		Shrinkage (%)			
			Neat PLA	PLA97 + J0.5/P0.1	PLA90 + J0.5/P0.1	PLA90 + J0.5/P0.2
MD	a	W	90.0 ± 23.2	93.3 ± 14.9	106.7 ± 18.3	100.0 ± 0.2
		L	105.4 ± 6.3	92.0 ±8.4	96.7 ± 7.5	95.1 ± 7.4
	b	W	92.7 ± 23.6	95.0 ± 11.2	107.0 ± 18.0	100.0 ± 0.1
		L	99.0 ± 0.1	93.8 ± 5.7	100.0 ± 0.1	93.2 ± 3.9
	c	W	93.0 ± 1.8	100.0 ± 0.1	106.7 ± 18.3	100.0 ± 0.1
		L	97.0 ± 10.9	98.0 ± 11.5	100.0 ± 0.1	96.5 ± 4.8
	d	W	100.0 ± 14.9	80.0 ± 44.7	107.0 ± 18.0	95.0 ± 11.2
		L	99.2 ± 6.7	98.3 ± 11.9	100.0 ±0.2	100.0 ± 9.1
TD	a	W	100.0 ± 13.8	56.0 ± 33.4	92.7 ± 1.9	81.3 ± 27.2
		L	100.0 ± 7.1	89.9 ±10.2	87.9 ± 3.5	86.7 ±10.9
	b	W	100.0± 14.9	51.7 ± 30.3	92.7 ± 1.5	75.3 ± 15.2
		L	100.0 ± 7.7	89.3 ± 7.9	89.3 ± 0.6	78.4 ± 26.2
	c	W	100.0 ± 14.2	45.0 ± 27.4	92.0 ± 1.8	80.3 ±18.7
		L	100.0 ± 9.0	89.3 ± 7.9	89.3 ± 0.8	81.8 ± 19.1
	d	W	98.3 ± 19.1	62.0 ±17.2	92.7 ± 1.8	76.7 ± 13.7
		L	106.9 ± 4.4	84.7± 12.8	90.2 ±5.4	89.9 ± 11.5

**Table 5:** The average percentage of shrinkage of film at a stretching ratio of 3 in MD and TD.

Direction	Position		Shrinkage (%)			
			Neat PLA	PLA97 + J0.5/P0.1	PLA90 + J0.5/P0.1	PLA90 + J0.5/P0.2
MD	a	W	63.3 ± 32.4	88.0 ± 26.8	92.9 ± 18.6	80.0 ± 28.3
		L	104.3 ± 6.1	87.5 ± 22.7	97.9 ± 2.8	80.1 ± 28.1
	b	W	82.3 ± 24.6	87.0 ± 18.6	108.7 ± 25.9	80.0 ± 24.5
		L	104.4 ± 6.1	83.5 ± 24.2	100.0 ± 0.1	83.7 ± 18.6
	c	W	79.1 ± 33.9	66.0 ± 29.0	117.0 ± 23.1	84.0 ± 26.1
		L	86.2 ± 4.2	67.9 ±23.2	82.5 ± 2.1	72.7 ± 13.7
	d	W	70.5 ± 39.1	84.0 ± 26.1	109.1 ± 18.6	79.0 ± 14.3
		L	103.4 ± 5.6	85.4 ± 24.3	99.0 ± 2.1	90.2 ± 10.2
TD	a	W	77.3 ± 24.4	62.0 ± 24.6	96.9 ± 3.0	80.0 ± 34.6
		L	90.4 ± 23.0	77.3 ± 17.0	95.1 ± 0.1	84.1 ± 30.3
	b	W	91.2 ± 32.2	83.0 ± 9.7	97.4 ± 2.9	75.0 ± 32.8
		L	92.8 ±17.1	91.0 ± 7.4	95.0 ± 0.2	82.1 ± 35.0
	c	W	82.4 ± 40.1	79.0 ± 2.2	97.4 ± 2.4	71.0 ± 30.1
		L	73.4 ± 43.9	77.6 ± 38.1	80.0 ± 2.2	69.2 ± 35.3
	d	W	91.3 ± 34.3	78.0 ± 2.7	97.6 ± 2.2	75.0 ± 32.8
		L	89.1 ± 34.1	94.0 ± 4.2	95.9 ±4.4	82.2 ± 40.2
